# Optimal Intravenous Administration Procedure for Efficient Delivery of Canine Adipose-Derived Mesenchymal Stem Cells

**DOI:** 10.3390/ijms232314681

**Published:** 2022-11-24

**Authors:** Yuyo Yasumura, Takahiro Teshima, Yoshiaki Taira, Takahiro Saito, Yunosuke Yuchi, Ryohei Suzuki, Hirotaka Matsumoto

**Affiliations:** 1 Laboratory of Veterinary Internal Medicine, Department of Veterinary Clinical Medicine, School of Veterinary Medicine, Faculty of Veterinary Science, Nippon Veterinary and Life Science University, 1-7-1 Kyonan-cho, Musashino, Tokyo 180-8602, Japan; 2 Research Center for Animal Life Science Nippon Veterinary and Life Science University, 1-7-1 Kyonan-cho, Musashino, Tokyo 180-8602, Japan

**Keywords:** adipose-derived mesenchymal stem cells, canine, cell delivery, cell viability, intravenous administration

## Abstract

Mesenchymal stem cells (MSC) are currently being investigated for their therapeutic applications in a wide range of diseases. Although many studies examined peripheral venous administration of MSC, few have investigated the detailed intravenous administration procedures of MSC from their preparation until they enter the body. The current study therefore aimed to explore the most efficient infusion procedure for MSC delivery by preparing and infusing them under various conditions. Canine adipose-derived mesenchymal stem cells (cADSC) were infused using different infusion apparatuses, suspension solutions, allogenic serum supplementation, infusion time and rates, and cell densities, respectively. Live and dead cell counts were then assessed by manual measurements and flow cytometry. Efficiency of live- and dead-cell infusion and cell viability were calculated from the measured cell counts and compared under each condition. Efficiency of live-cell infusion differed significantly according to the infusion apparatus, infusion rate, and combination of cell density and serum supplementation. Cell viability after infusion differed significantly between the infusion apparatuses. The optimal infusion procedure resulting in the highest cell delivery and viability involved suspending cADSC in normal saline supplemented with 5% allogenic serum at a density of 5 × 10^5^ cells/mL, and infusing them using an automatic infusion device for 15 min. This procedure is therefore recommended as the standard procedure for the intravenous administration of ADSC in terms of cell-delivery efficiency.

## 1. Introduction

Mesenchymal stem cells (MSC) possess self-renewal and multilineage potentials, and also demonstrate tissue repair, angiogenesis, and immunomodulatory effects. MSC are currently being investigated for their therapeutic applications in a wide range of diseases, including neurological disorders [1] and osteoarticular [2], cardiac [3], and immune-mediated diseases [4]. According to the minimal criteria defined by the International Society for Cell & Gene Therapy, MSC are cells that are positive for CD73, CD90, and CD105 expression and negative for hematopoietic markers such as CD14, CD19, CD34, CD45, CD79, have the osteogenic, chondrogenic and adipogenic differentiation potential in vitro, and have adhesive properties to plastics [5]. Cells meeting this criterion have been isolated from a variety of human tissues to date, since the first bone marrow-derived MSC (BMSC) were isolated from mice [6]. BMSC and adipose-derived MSC (ADSC) are the most commonly studied MSC types, but MSC from many other tissue sources, including umbilical cord, dental pulp, placenta, skin, and peripheral blood have also been isolated and researched [7]. MSC derived from different tissues share many common characteristics, but differ in proliferative capacity, growth factor secretion, immunomodulatory capacity, and differentiation capacity, and their properties vary depending on culture conditions [4,8]. In addition, there are differences in the difficulty of collection and the invasiveness to the donor depending on the tissue [9]. Therefore, for therapeutic application of MSC, it is desirable to select a tissue source that facilitates the process of collection, isolation, and expansion and has more favorable biological properties for the disease [10].

Among a number of different tissue-derived MSC, human BMSC (hBMSC), human ADSC (hADSC), and human umbilical cord MSC (hUCMSC) are the most commonly used human MSC tissue sources in clinical trials [11]. Bone marrow was the first established tissue source of MSC [6], and hence BMSC are the most studied MSC type. hBMSC have anti-inflammatory, immunomodulatory, and tissue repair effects through differentiation into damaged tissue cells via their multipotency and secretion of soluble factors, and have been used most frequently in preclinical and clinical trials, with promising results reported with few side effects in the treatment of various diseases [12]. However, hBMSC have the disadvantages of being more invasive than other tissue sources because they require bone marrow puncture in collection [13] and require expanded culture due to low cell yield, which may decrease cell quality [14,15]. On the other hand, adipose tissue contains 100–1000 times more MSC than bone marrow and is known to be the major tissue source of MSC [16]. hADSC are easier to collect and isolate than BMSC and are the second most commonly applied clinically after hBMSC [17]. Although the chondrogenic and osteogenic potential of hADSC is inferior to that of hBMSC [13], it has been reported that the secretory capacity of growth factors and immunomodulatory factors is higher than that of hBMSC, and that their tissue repair and immunomodulatory capacities are higher [8,18]. Therefore, the safety and simplicity of collection and the functional advantages make hADSC highly feasible for clinical applications. The umbilical cord, a perinatal appendage, is also rich in MSC and has attracted attention as a novel tissue source of allogeneic MSC because it is not physically invasive to the donor [19]. hUCMSC have high proliferative, immunomodulatory, and tissue repair capacity in vitro, and unlike BMSCs, they are considered to have low immunogenicity because human lymphocyte antigen-DR is not induced even in the presence of the inflammatory cytokine INF-γ [20,21,22]. It has also been reported that hUCMSC have a higher capacity to differentiate into the nervous system and are more neuroprotective than hBMSC [23,24]. hUCMSC is also undergoing clinical trials following hBMSC and hADSC, and its beneficial effects have been reported [25]. Dental pulp-derived MSC (DPSC) are considered to be one of the attractive sources of MSC tissue because they can be harvested in conjunction with tooth extraction, which is part of routine dental procedures, without additional invasiveness to the donor [26]. Human DPSC have potential therapeutic applications in orthopedics, oral and maxillofacial reconstruction, and nerve repair [27,28]. MSC derived from the various tissue sources described above have shown efficacy in intravenous administration for various diseases, e.g., graft-versus-host disease [29,30,31], but it is still controversial which tissue source-derived MSC are most suitable for therapeutic application for each disease [32,33].

MSC have been isolated from a number of tissue sources in veterinary medicine, including bone marrow, adipose tissue, umbilical cord, and dental pulp, etc [34]. The main tissue sources most commonly studied in dogs are bone marrow and adipose tissue, as in humans, but adipose tissue is particularly attractive because it is can be harvested during routine sterilization with low donor invasion, has greater proliferative potential than canine BMSC (cBMSC), and can expand easily [35,36]. Indeed, therapeutic applications of MSC have been reported in a variety of canine diseases, including orthopedic, neurological, gastrointestinal, cardiac, ophthalmic, and dermatological disorders, many of which use intravenous administration of canine ADSC (cADSC) [37].

Although many studies have reported promising results with MSC, the appropriate source of MSC, number of cells, route and timing of administration, and patient indications remain unclear. The optimal route of administration of hADSC for different diseases is still unknown, but many studies have chosen the peripheral venous administration route [38]. With respect to intravenous administration, information on the in vivo behavior of the cells has been partially obtained via in vivo imaging of fluorescently or radioisotope-labeled hADSC [39]. In addition, the effects of different cell preparations, manufacturing and storage protocols, and administration methods on cell viability, phenotype, and function have been investigated in human MSC [40,41]. However, few reports have examined the detailed intravenous administration procedures of ADSC from their preparation until they enter the body. For cellular therapy, it is important that the intended number of cells is delivered into the patient as completely and intact as possible, to ensure reproducible therapeutic efficacy and in order to assess the validity of the treatment plan. The current study thus aimed to explore the most efficient infusion procedure for the delivery of ADSC into patients by comparing the delivery efficiency of cells prepared and infused under various conditions in vitro. The best cell-delivery efficiency was achieved by infusion cADSC under selected conditions with higher efficiency of live-cell infusion and cell viability. We expected that the findings of this study could contribute to standardizing the intravenous administration of ADSC therapy, thereby facilitating the comparison of results across studies and achieving consistent therapeutic effects.

## 2. Results

### 2.1. Characterization of cADSC

**Figure 1:**
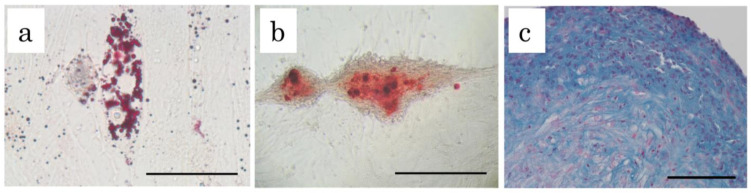
Differentiation of cADSC into three lineages. (**a**), Adipogenic differentiation identified by Oil Red O staining; (**b**), Osteogenic differentiation identified by Alizarin Red staining; (**c**), Chondrogenic differentiation identified by Alcian Blue. Bar = 100 μm.

cADSC were successfully cultured and expanded. Most of the cells expressed the MSC surface markers CD29 (97.48 ± 0.66%), CD44 (98.56 ± 0.70%), and CD90 (97.18 ± 0.16%), and few expressed CD14 (0.34 ± 0.06%), CD34 (0.26 ± 0.04%), and CD45 (0.30 ± 0.07%). The cADSC exhibited multipotency, as demonstrated by their potential for adipogenic, osteogenic, and chondrogenic differentiation (Figure 1).

### 2.2. Evaluation of Cell Delivery and Cell Viability under Each Infusion Condition

#### 2.2.1. Infusion Apparatus

**Figure 2:**
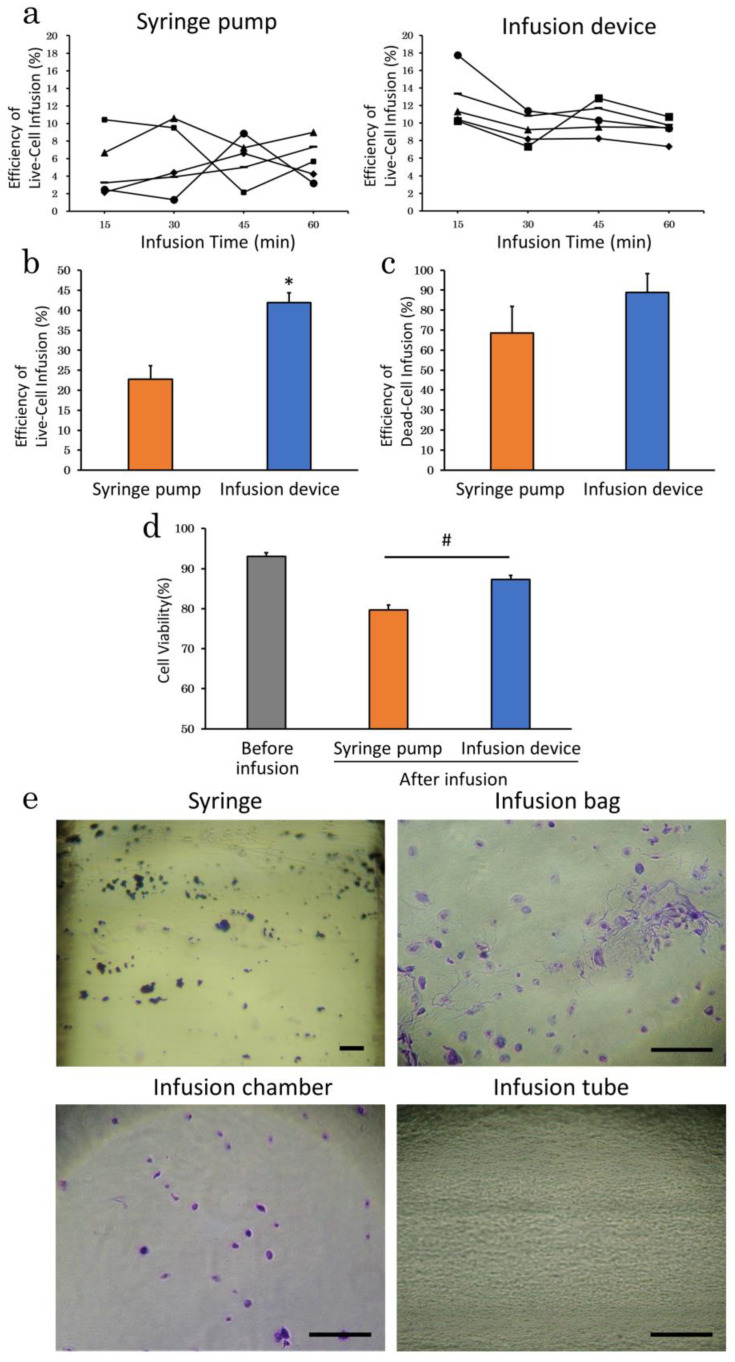
Infusion apparatus experiments. (**a**), Efficiency of live-cell infusion every 15 min using syringe pump (**left** panel) and infusion device (**right** panel). Each broken line represents the results of five independent experiments (*n* = 5); (**b**), Efficiency of live-cell infusion over 60 min. The infusion device increased efficiency of live-cell infusion approximately two-fold compared with the syringe pump; (**c**), Efficiency of dead-cell infusion over 60 min; (**d**), Cell viability after 60 min infusion. The cell viability using an infusion device was significantly higher than using a syringe pump; (**e**), Adherent cells in the apparatuses stained by Giemsa staining. cADSC adhered to various parts of the infusion apparatuses. No cell adhesion was observed in the tubes. * *p* < 0.01 vs. syringe pump. # *p* < 0.01, between groups. Bar = 200 μm.

To determine the effect of the infusion apparatus on cell delivery, cADSC were infused using an automatic infusion device or syringe pump. Based on cell efficiency of live-cell infusion per 15 min, the infusion devices showed a gradual decrease over time, while syringe pumps showed no consistent trend (Figure 2a). Efficiency of live-cell infusion over 60 min was significantly higher with the infusion device (41.9 ± 2.5%; *p* < 0.01) compared with the syringe pump (22.8 ± 3.4%) (Figure 2b), but there was no significant difference in efficiency of dead-cell infusion (88.9 ± 9.4%; 68.5 ± 13.3%, respectively; *p* = 0.24) (Figure 2c). Cell viability after infusion was significantly higher with the infusion device (87.3 ± 1.0%; *p* < 0.01) than with the syringe pump (79.7 ± 1.3%) (Figure 2d). We therefore used an infusion device in all subsequent experiments.

To visualize cell adhesion in the apparatuses, the infusion bag with an infusion tube or syringe with an extension tube was fixed by filling with 10% formalin immediately after infusion. After 10 min, the formalin was poured off and replaced with Giemsa stain and incubated for 10 min to stain adherent cADSC. Adherent cADSC for both apparatuses were then observed under a microscope (Figure 2d). Cell adhesion was mostly observed on the walls of the infusion bag, syringe, and drip chamber, but not on the infusion tube or extension tube.

#### 2.2.2. Suspension Solution

**Figure 3:**
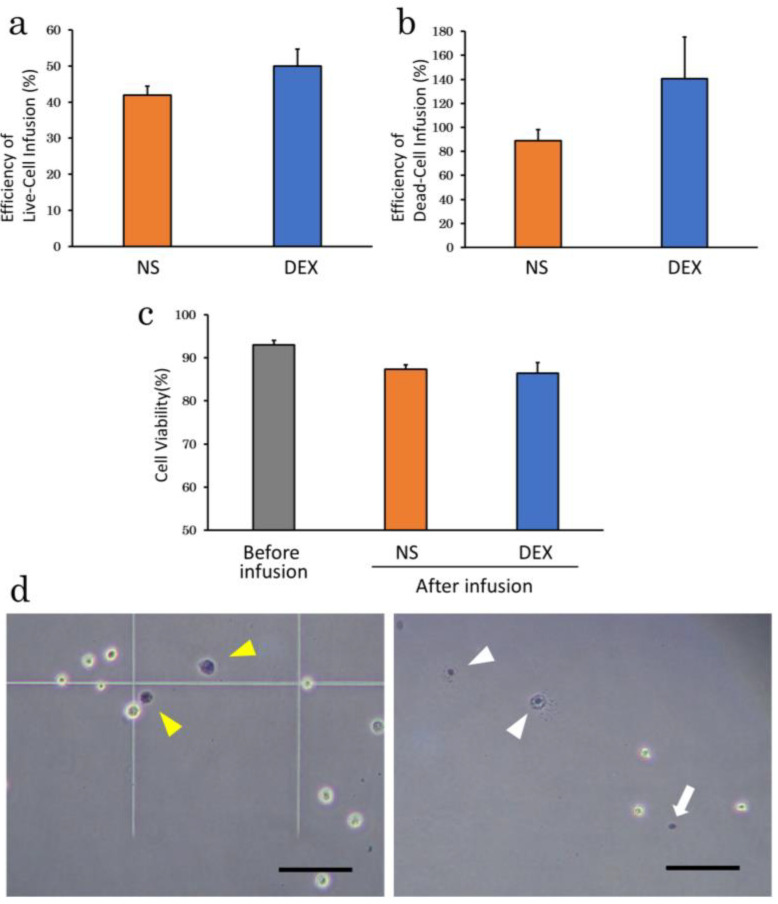
Efficiency of cell infusion and cell viability in different suspension solutions. (**a**), Efficiency of live-cell infusion for NS and DEX. There were no significant differences, but efficiency of live-cell infusion tended to be higher in DEX; (**b**), Efficiency of dead-cell infusion for NS and DEX. Efficiency of dead-cell infusion also tended to be higher in DEX; (**c**), Cell viability after infusion for NS and DEX showed a slightly lower in DEX; (**d**), Microscope images of dead cADSC suspended in NS (**left** panel) or DEX (**right** panel). Trypan blue-stained dead cells suspended in NS were comparable in size and morphology to live cells (**left** panel; yellow arrowheads). Small concentrated Trypan Blue-stained dead cells (**right** panel; white arrows) and cells that appeared to be bursting (**right** panel; white arrowheads) were observed in DEX. NS: normal saline; DEX: 5% dextrose. Bar = 100 μm.

Efficiency of cell infusion differed following infusion of cADSC under basal condition using the two different suspension solutions. Efficiency of live-cell infusion tended to be higher with 5% dextrose solution (DEX; 50.0 ± 4.7%) than 0.9% normal saline (NS; 41.9 ± 2.5%), although the difference was not significant (*p* = 0.16) (Figure 3a). Efficiency of dead-cell infusion also tended to be higher in DEX (140.7 ± 34.5%) than in NS (88.9 ± 9.4%; *p* = 0.18), with the DEX exceeding 100% (Figure 3b). On the other hand, cell viability after infusion tended to be slightly lower for cells in DEX (NS, 87.3 ± 1.0%; DEX, 86.4 ± 2.4%; *p* = 0.75) (Figure 3c). Microscopically, Trypan Blue-stained cells appeared smaller and more concentrated in DEX (Figure 3d, right panel), with signs of ‘‘bursting’’ cells.

#### 2.2.3. Serum Supplementation

**Figure 4:**
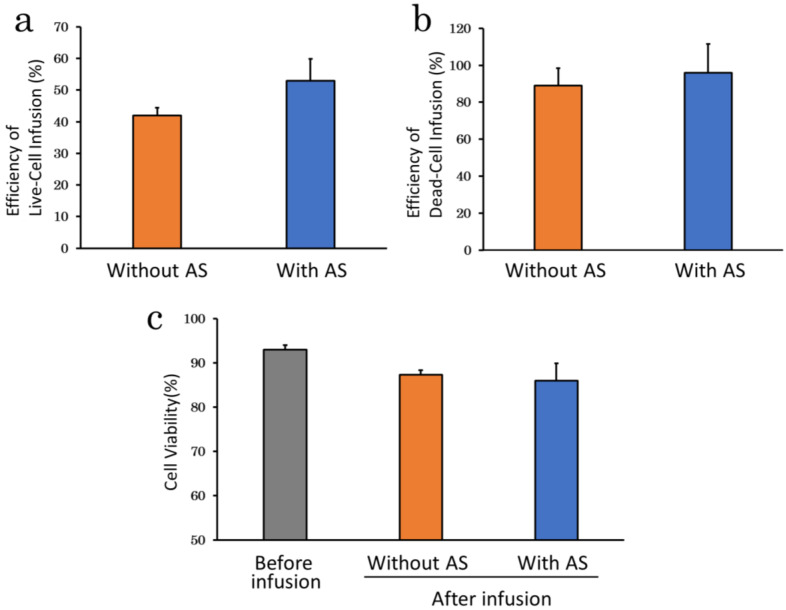
Differences in efficiency of cell infusion and cell viability with and without AS. (**a**), Efficiency of live-cell infusion with and without AS. There were no significant differences, but efficiency of live-cell infusion tended to be higher with AS; (**b**), Efficiency of dead-cell infusion with and without AS. Efficiency of dead-cell infusion also tended to be higher with AS; (**c**), Cell viability with and without AS. Supplementation of AS slightly decreased in viability. AS: allogenic serum.

Efficiency of cell infusion was compared between cells suspended with and without 5% canine allogenic serum (AS) under basal condition to confirm the effect of serum supplementation on cell delivery efficiency. Efficiency of live-cell infusion tended to be higher with AS (53.0 ± 6.9%) than without AS (41.9 ± 2.5%), although the difference was not significant (*p* = 0.18) (Figure 4a). Efficiency of dead-cell infusion also tended to be higher with (96.0 ± 15.5%) than without AS (88.9 ± 9.4%; *p* = 0.53) (Figure 4b) and the cell viability after infusion tended to be slightly lower with (86.0 ± 3.9%) than without AS (87.3 ± 1.0%; *p* = 0.76) (Figure 4c).

#### 2.2.4. Infusion Time

**Figure 5:**
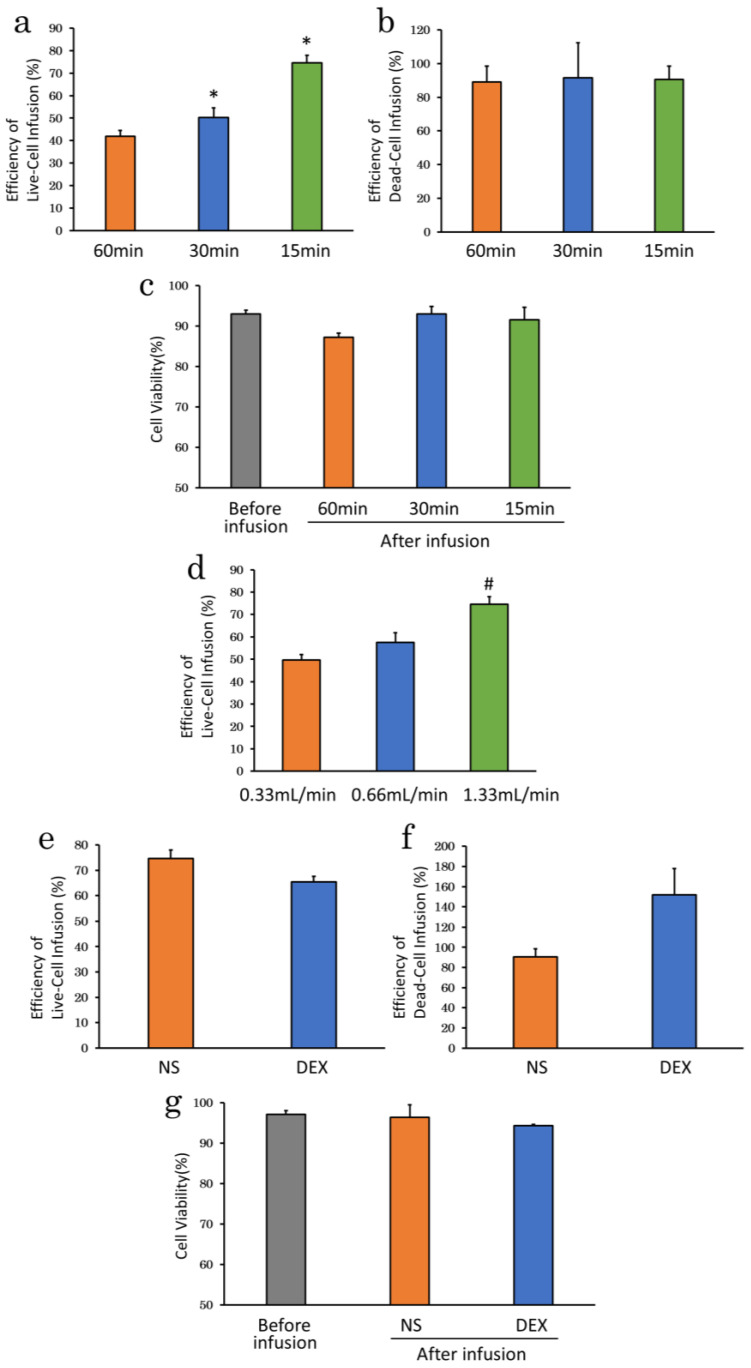
Infusion time experiments. (**a**), Efficiency of live-cell infusion for 15, 30, and 60 min infusions. Efficiency of live-cell infusion was significantly higher for 15 and 30 min than for 60 min; (**b**), Efficiency of dead-cell infusion did not differ among the three time groups; (**c**), Cell viability after infusion tended to be higher at 15 and 30 min; (**d**), Differences in efficiency of live-cell infusion by infusion rate. Faster infusion rates tended to result in higher efficiency of live-cell infusion; (**e**), Efficiency of live-cell infusion after 15 min infusion with different suspension solutions. In contrast to infusion over 60 min, efficiency of live-cell infusion tended to be better in NS; (**f**), Efficiency of dead-cell infusion after 15 min infusion with different suspension solutions. Efficiency of dead-cell infusion was high at DEX, exceeding 100%; (**g**), Cell viability after 15 min infusion with different suspension solutions. * *p* < 0.01 vs. 60 min. # *p* < 0.05 vs. 0.33 mL/min. NS: normal saline; DEX: 5% dextrose.

We divided the time from the start to the end of infusion into three groups (15, 30, and 60 min) under basal condition and investigated the effect of infusion time on cell delivery. A shorter infusion time improved efficiency of live-cell infusion, with significant differences between 15 (74.6 ± 3.4%; *p* < 0.01) and 30 min (50.4 ± 4.2%; *p* < 0.01) compared with 60 min infusion (41.9 ± 2.5%) (Figure 5a). In contrast, there was no difference in efficiency of dead-cell infusion among the three groups (15 min, 90.5 ± 8.0%; 30 min, 91.5 ± 20.9%; 60 min, 88.9 ± 9.4%; *p* = 0.99) (Figure 5b). There was also no significant difference in cell viability after infusion among the three groups (*p* = 0.09), but it tended to be higher for 15 (91.6 ± 3.1%) and 30 min (93.0 ± 1.9%) than for 60 min (87.3 ± 1.0%) (Figure 5c).

The suspension volume and cell densities were identical for all three infusion times, and the dosing rates thus differed among the groups (15 min, 1.33 mL/min; 30 min, 0.67 mL/min; 60 min, 0.33 mL/min). We therefore infused the cells for 15 min at three different infusion rates to compare the effects of infusion rate on cell delivery. A faster infusion rates improved efficiency of live-cell infusion, with significant difference between 1.33 mL/min (74.6 ± 3.4%; *p* < 0.01) compared with 0.33 mL/min group (49.6 ± 6.0%), but there was no significant difference between 0.66 mL/min (57.6 ± 5.2%; *p* = 0.08) and 0.33 mL/min group (Figure 5d).

Infusion of cADSC suspended in NS for a short time at a fast-infusion rate resulting in high efficiency of live-cell infusion. We therefore performed the same experiment with DEX, which had a higher efficiency of live-cell infusion for 60 min. Efficiency of dead-cell infusion was not affected by 15 min infusion (151.8 ± 26.1%), but efficiency of live-cell infusion (65.4 ± 2.2%) and cell viability after infusion were improved (94.4 ± 0.2%) compared with 60 min (efficiency of dead-cell infusion, 140.7 ± 34.5%; *p* = 0.80; efficiency of live-cell infusion, 50.0 ± 4.7%; *p* < 0.05; cell viability, 86.4 ± 2.4%; *p* < 0.05). However, compared with the 60 min infusion, 15 min infusion tended to result in higher efficiency of live-cell infusion in NS (NS, 74.6 ± 3.4%; DEX, 65.4 ± 2.2%; *p* = 0.05) (Figure 5e). Efficiency of dead-cell infusion tended to be lower in NS (*p* = 0.11) (Figure 5f), and cell viability after 15 min of infusion tended to be higher in NS (*p* = 0.63) (Figure 5g).

#### 2.2.5. Cell Density

**Figure 6:**
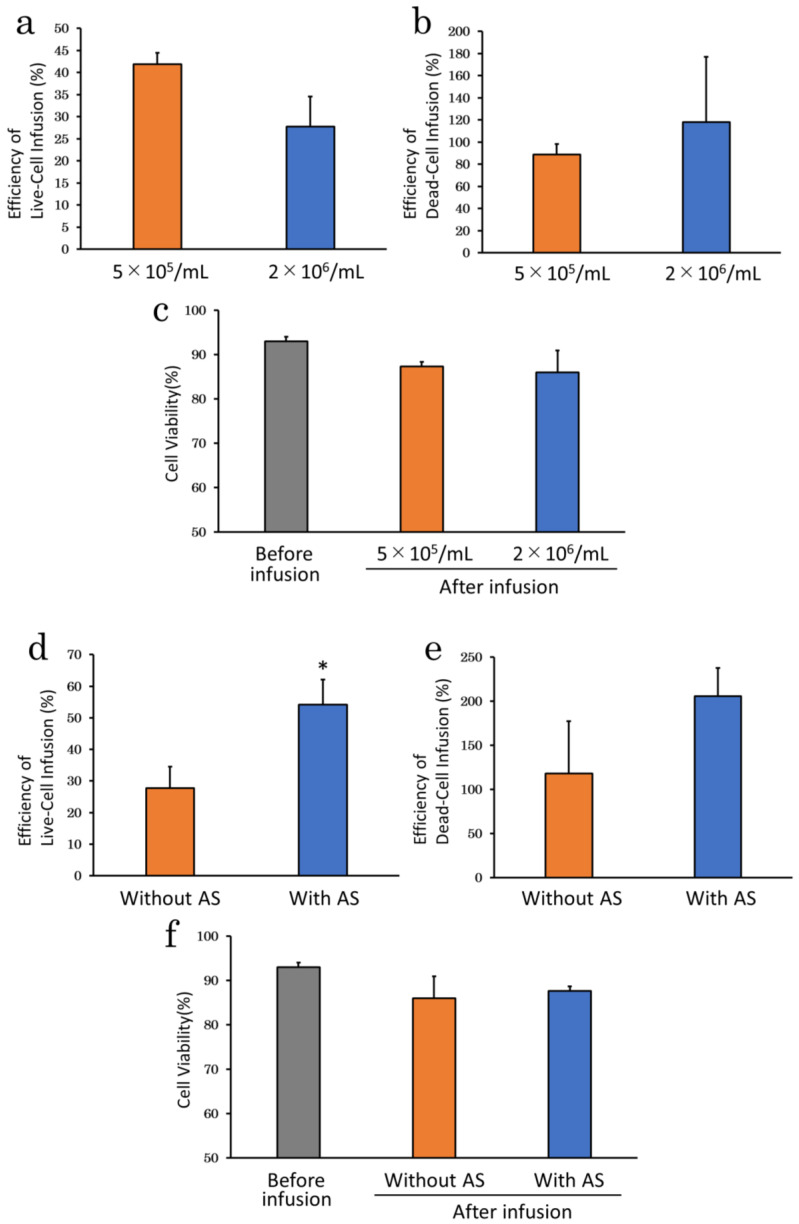
Cell density experiments. (**a**), Efficiency of live-cell infusion at different cell densities. Higher cell density tended to result in lower efficiency of live-cell infusion; (**b**), Efficiency of dead-cell infusion at different cell densities. Higher cell density tended to result in higher efficiency of dead-cell infusion; (**c**), Cell viability at different cell densities. Higher cell density resulted in lower cell viability after infusion; (**d**), Effect of serum supplementation at high cell density. AS supplementation significantly improved efficiency of live-cell infusion at high cell density; (**e**), Effect of serum supplementation on efficiency of live-cell infusion at high cell densities; (**f**), Differences in cell viability after infusion with and without serum supplementation at high cell densities. * *p* < 0.05 vs. without AS. AS: allogenic serum.

We prepared cADSC at two cell densities (5 × 10^5^ and 2 × 10^6^ cells/mL) and infused them for 60 min (0.33 mL/min) under basal condition to investigate the effect of cell density. Efficiency of live-cell infusion tended to be lower in the 2 × 10^6^ cells/mL (27.8 ± 6.8%; *p* = 0.08) than in the 5 × 10^5^ cells/mL group (41.9 ± 2.5%) (Figure 6a), while efficiency of dead-cell infusion tended to be higher in the 2 × 10^6^ cells/mL (118.0 ± 59.2%; *p* = 0.64) than in the 5 × 10^5^ cells/mL group (88.9 ± 9.4%) (Figure 6b). Cell viability after infusion tended to be lower for 2 × 10^6^ cells/mL (86.0 ± 4.9%; *p* = 0.81) than for 5 × 10^5^ cells/mL group (87.3 ± 1.0%) (Figure 6c).

We further investigated the effect of the combination of cell density and serum supplementation on cell delivery, given that the effect of serum supplementation was not fully demonstrated in the previous experiment and efficiency of live-cell infusion decreased at high cell density. cADSC were prepared at a density of 2 × 10^6^ cells/mL and supplemented with 5% AS, and infused for 60 min. Supplementation of 5% AS mildly increased efficiency of dead-cell infusion (206.0 ± 31.8%; *p* = 0.27). However, unlike at low cell density, supplementation of 5% AS at high cell density significantly improved efficiency of live-cell infusion (54.2 ± 7.8%; *p* < 0.05) and tended to increase cell viability after infusion (87.6 ± 1.1%; *p* = 0.76) (Figure 6f).

### 2.3. Evaluation of Cell Viability by Flow Cytometry

**Figure 7:**
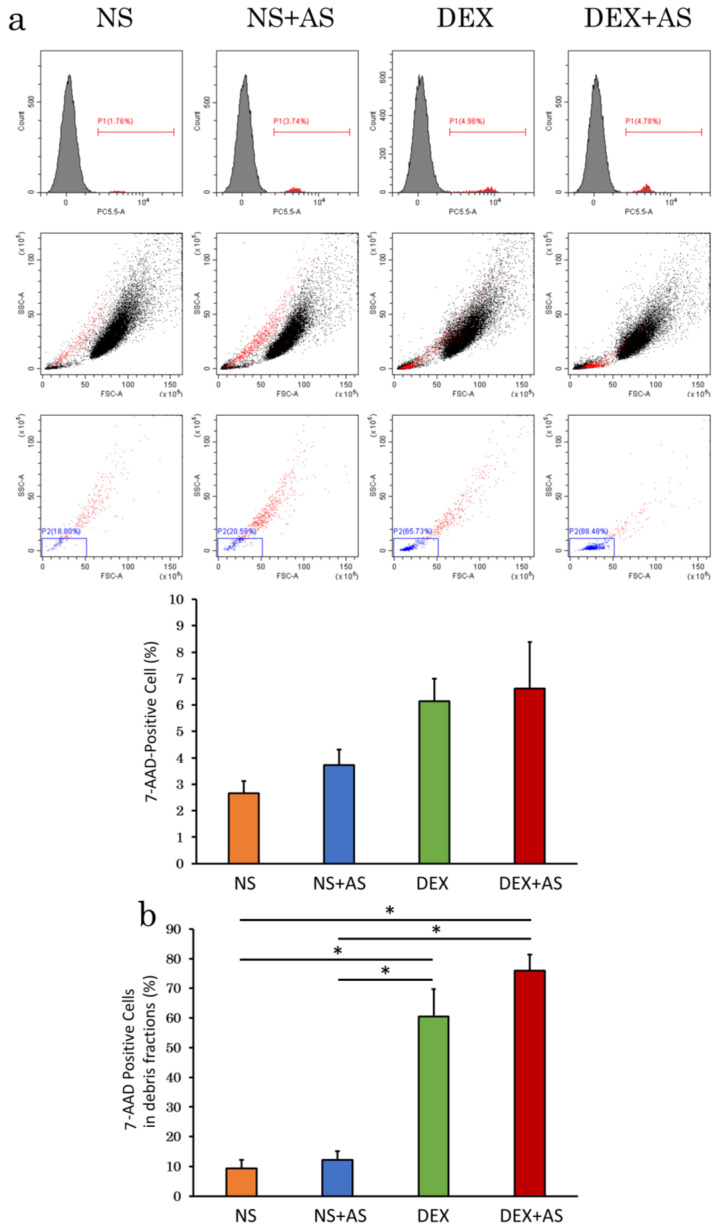
Flow cytometry analysis of dead cells stained with 7-AAD. (**a**), Dot plots and histograms of all analyzed cells (**top** and **middle** rows), and dot plots of 7-AAD-positive cells gated by debris fraction (**lower** row). Although there was no significant difference, percentage of 7-AAD-positive cells were lower with NS than with DEX, and without than with AS (*p* = 0.05); (**b**), Suspension in DEX significantly increased 7-AAD-positive cells in the debris fraction. * *p* < 0.01, between groups. NS: normal saline; AS: allogenic serum; DEX: 5% dextrose.

Flow cytometric analysis of cADSC incubated for 30 min under four different preparation conditions showed 7-amino-actinomycin D (7-AAD)-positive cells. There was no significant difference in the 7-AAD-positive cell rate between the four preparation conditions (NS, 2.7 ± 0.5%; NS + AS, 3.7 ± 0.6%; 5% dextrose, 6.1 ± 0.9%; DEX + AS, 6.6 ± 1.8%; *p* = 0.05) (Figure 7a). Although all conditions showed adequate cell viability (>90%), viability tended to be better in cells suspended in NS compared with DEX, and in cells without serum compared with those with serum (NS, 97.3 ± 0.5%; NS + AS, 96.3 ± 0.6%; DEX, 93.9 ± 0.9%; DEX + AS, 93.4 ± 1.8%; *p* = 0.05). When cADSC were suspended in DEX, 7-AAD-positive cells significantly increased in the very small debris fractions (NS, 9.4 ± 2.8%; NS + AS, 12.2 ± 3.0%; DEX, 60.6 ± 9.2%; DEX + AS, 75.9 ± 5.5%; *p* < 0.01) (Figure 7b).

### 2.4. Assessment of Optimized Infusion Procedures

**Figure 8:**
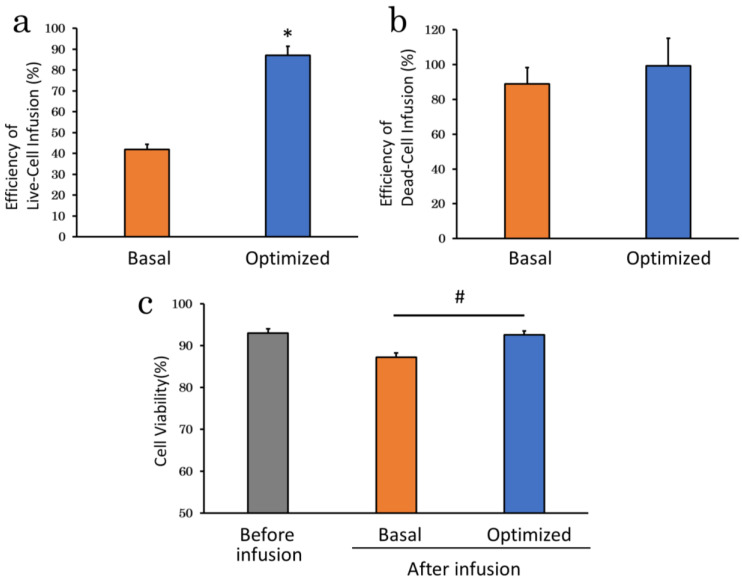
Optimized infusion procedure. (**a**), Efficiency of live-cell infusion under optimized conditions. The optimized infusion procedure significantly improved efficiency of live-cell infusion compared with the basal condition, which were the best results in this study; (**b**), Efficiency of dead-cell infusion of infusion under optimized conditions. Efficiency of dead-cell infusion was slightly higher than the basal condition; (**c**), Cell viability of infusion under optimized conditions. The optimized infusion procedure significantly improved cell viability after infusion compared with the basal condition. * *p* < 0.01 vs. control. *# p* < 0.01, between groups.

Considering the results of the above experiments, we selected the most suitable infusion apparatus, suspension solution, serum supplementation, infusion time and rate, and cell density. Specifically, cADSC were suspended in NS supplemented with 5% AS at a density of 5 × 10^5^ cells/mL and then infused using an automatic infusion device for 15 min (1.33 mL/min). Infusion under these optimized conditions mildly increased efficiency of dead-cell infusion (99.2 ± 15.9%; *p* = 0.29) compared with the basal condition (88.9 ± 9.4%) (Figure 8b), but significantly improved efficiency of live-cell infusion (basal vs. optimized: 41.9 ± 2.5% vs. 87.0 ± 4.4%; *p* < 0.01) (Figure 8a) and cell viability after infusion (basal vs. optimized: 87.3 ± 1.0% vs. 92.6 ± 0.9%; *p* < 0.01) (Figure 8c). Efficiency of live-cell infusion and cell viability after infusion were highest throughout all experiments.

## 3. Discussion

We hypothesized three possible causes of poor cell-delivery efficiency during intravenous infusion: (1) acute cell death due to nutrient deprivation and hypoxic stress in the suspension solution or biomechanical stress during infusion; (2) cell adhesion to the walls of the apparatus, such as the infusion bag, syringe, or infusion tube; and (3) cell aggregation during storage and infusion. Temperature, solution type, and cell density of the cell preparation, as well as storage, can affect the metabolic activity of MSC, thus altering their viability and function [42,43,44,45,46,47,48,49,50,51]. It has been reported that the viability of hADSC in NS is better maintained at lower temperatures (4 °C) than at room temperature (37 °C) [42,43]. On the other hand, Kubrova et al. compared the viabilities of hADSC suspended in lactate Ringer’s solution and stored at physiological temperature (37 °C), room temperature (23 °C), or low temperature (4 °C) for 4 h, and reported that room temperature was optimal [44]. Because the current study focused on cell delivery in infused ADSC immediately after preparation, we considered the effect of temperature-related acute cell death to be minimal due to their short storage time, and we therefore prepared the cADSC at room temperature in this study. Indeed, 7-AAD staining after 15 min of incubation at room temperature confirmed sufficient cell viability in either solution.

Biomechanical stresses, such as ejection pressure and shear stress during cell infusion through a small-bore needle, syringe, or catheter, can result in immediate cell death or delayed apoptosis [52,53,54,55,56,57]. The ejection pressure and shear stress applied during cell injection are defined by the density and viscosity of the cell suspension, the infusion rate, and the size of the needle, syringe, or catheter, with lower density and viscosity, slower infusion rate, and larger needle resulting in lower biomechanical forces [52]. Studies examining the effect of passage through several bore size needles on the viability of hADSC [53] and hBMSC [54] failed to detect immediate cell death or delayed apoptosis after passage through small-bore needles. On the other hand, human bone marrow mononuclear cells showed decreased viability immediately after passage through the needle and catheter at an infusion rate of 5 mL/min, but were unaffected at infusion rates of 2 mL/min or less [55]. Walker et al. also reported that passage of rat BMSC and hBMSC through needles and catheters did not cause an immediate decrease in viability [56]. The needle bore (bore, 0.5 mm; length, 16 mm), infusion tube (bore, 2.4 mm; length, 1250 mm), and extension tube (bore, 2.1 mm; length, 750 mm) used in the present study were smaller than those in previous studies, the infusion rate (1.33 mL/min) did not cause immediate cell death, and the cell density and solution viscosity were also low. It is therefore unlikely that these infusion conditions caused biomechanical forces that could have resulted in reduced cell viability immediately after infusion. In fact, although efficiency of dead-cell infusion was consistently higher than of live-cell infusion under all conditions, efficiency of dead-cell infusion did not exceed 100% except under conditions of DEX suspension and high cell density, and cell viability also decreased by less than 10% from before to after infusion throughout the experiment, with the exception of the syringe pump infusion group. Taken together, although cell-delivery efficiency was partially affected by acute cell death in some conditions, most of the difference in cell-delivery efficiency was attributed to cell adhesion to the infusion apparatus and aggregation of cells with each other. Adhesion of cADSC to the apparatus wall was confirmed by Giemsa staining immediately after infusion.

Intravenous administration is the most common route for administering MSC to patients [38]. However, some studies have used manual or syringe pumps to administer MSC intravenously for therapeutic purposes [58,59,60] while others used infusion bags [61,62,63], and clear criteria for their choice have not been established. This study thus compared the suitabilities of two apparatuses for ADSC administration. The results showed that infusion of cADSC using an automatic infusion device resulted in significantly better efficiency of live-cell infusion and cell viability compared with a syringe pump. The syringe pump was set horizontally for infusion in this study. Although a horizontal syringe is less susceptible to cell sedimentation than a vertical syringe, cell suspensions with lower cell volume fractions and lower viscosity are more susceptible to sedimentation [53]. Under the current study conditions, the viscosity of the NS used for suspension was low [64] and the cell volume fraction was very low (0.02%), and the longer infusion time may have resulted in sedimentation and adhesion of cADSC in the syringe, whereas the automatic infusion device would have minimized the loss of efficiency of live-cell infusion due to sedimentation, because the direction of gravity-induced sedimentation coincided with the direction of cell delivery. In addition, the mismatch between the direction of cell sedimentation and the direction of delivery may have resulted in variations in the distribution of cells in the suspension, which could affect cell delivery. These findings suggest that the increased number of cells adhering to the syringe wall and increased cell aggregation may have affected the low delivery efficiency associated with use of the syringe pump. Both efficiency of live- and dead-cell infusion were higher with the infusion device, but the ratio of efficiency of dead-cell infusion to of live-cell infusion was higher with the syringe pump. Dead cells are known to lose their ability to adhere: a syringe pump thus does not increase acute cell death, but rather recovers more dead cells that have lost adhesion than live cells. Delivery efficiency may be improved by using a vertical syringe pump or by increasing the density and viscosity of the cell suspension; however increased shear stress may result in cell damage. We therefore recommend using an infusion device for infusion of ADSC. Following these results, we performed our subsequent experiments using an infusion device.

Various injection solutions, mainly NS, have been used in clinical trials of MSC therapy [40]. Some commercial ADSC products are prepared with DEX, and clinical studies using this suspension solution have been reported [65,66]. Accordingly, we compared the cell delivery efficiencies of NS and DEX as suspension solutions. The viability tended to be lower while the efficiency of live-cell infusion tended to be higher in DEX, and Trypan Blue staining revealed small concentrated dead cells under an inverted microscope. Similarly, flow cytometry analysis confirmed an increase in 7-AAD-positive cells in the very small debris fractions. Suspension in DEX appeared to cause cell bursting and an increase in dead cells. Pal et al. reported that DEX was suitable for preserving hBMSC [45], but similar studies with hUCMSC [46], hADSC [47], and cADSC [48] reported low viability with this solution. The mechanism responsible for the decreased viability is unknown, but may be related to factors other than pH or osmotic pressure [46]. Based on efficiency of live-cell infusion, it is possible that DEX reduced cell aggregation and adhesion, but similar efficiency of live-cell infusion was obtained with NS at a high infusion rate. We therefore believe that cells should be suspended in NS. However, recent studies reported high viability of hADSC in lactate Ringer’s solution, which contains calcium and potassium ions and more closely resembles the electrolyte composition of body fluids [43,44]. Furthermore, the presence of calcium has been reported to reduce cell aggregation in hBMSC [49]. Further experiments with lactate Ringer’s solution are therefore needed.

Supplementation with human serum or serum albumin has been used to protect cells from environmental stresses and prevent them from adhering to the walls of tubes and needles [40,67,68,69]. We therefore expected that AS supplementation would improve cell viability by inhibiting cell adhesion to the apparatus. Efficiency of live-cell infusion accordingly tended to improve with AS supplementation, and the effect was more pronounced at high cell density. Albumin prevents cells from adhering to the material surface [70]. However, an evaluation of rat BMSC suspended in NS by flow cytometry reported no increase in cell aggregation in relation to cell density or time course [50]. These findings suggest that AS supplementation may have inhibited the adhesion of cADSC to the apparatus rather than inhibiting their aggregation. In the presence of serum, cells adhere to the material surface via integrin–extracellular matrix-mediated adhesion, while adhesion occurs via a different mechanism under serum-free conditions, and this process is inhibited by serum [71]. Further studies could clarify the contribution of cell adhesion to cell delivery by adding arginine-glycine-aspartic acid (RGD) peptides [72], which inhibit integrin binding to the extracellular matrix, simultaneously with AS. Conversely, AS supplementation tended to decrease cell viability. Suspension of hBMSC with NS containing 5% albumin was reported to maintain high cell viability [51]. However, the current study used non-heat-inactivated AS rather than purified albumin. It has been reported that contact with serum leads to complement activation and destruction of hBMSC [73]. The reduction in cell viability due to AS observed in the present study may be related to the destruction of cADSC by antibodies and complement contained in the AS. This mechanism needs further investigation using heat-inactivated serum, autologous serum, and purified albumin.

Intravenous administration of MSC has been performed over a range of times [41], including 15 min [61], 30 min [62], and more than 1 h [63,74]. The current study compared efficiency of cell infusion among infusions of 15, 30, and 60 min, and showed that a shorter infusion time led to better efficiency of live-cell infusion. Similar results were obtained for efficiency of live-cell infusion at different infusion rates. A faster infusion rate and shorter infusion time thus avoided cell adhesion and aggregation and improved efficiency of live-cell infusion. In detail, a fast infusion rate inhibited cell residence by creating a continuous fluid, while a short infusion time reduced the time available for cells to settle, adhere, and aggregate, which may have led to improved efficiency of live-cell infusion. Furthermore, adherent cells were not observed in narrow tubes, supporting the idea that continuous fluid flow inhibited cell adhesion to the wall. Although the cell viability after infusion tended to be higher in the 60 min infusion group, there was no difference in efficiency of dead-cell infusion between the infusion-time groups, meaning that long infusion times reduce the efficiency of live-cell infusion. This result also supports the idea that a short-infusion time may avoid the adhesion of viable cells to the apparatus. In summary, the fastest possible infusion rate and shortest possible infusion time allowed by the patient’s underlying disease should be used to maximize cell-delivery efficiency. However, the rapid administration of large amounts of cells can cause cell embolization and an immediate blood-mediated inflammatory response [75], and further research is therefore needed to establish safe dosing and dosage regimens.

Prior to administration, MSC are conditioned at various densities [61,74], and cell density has been reported to affect the properties of human and horse MSC, including their viability, proliferative capacity, and differentiation potential [47,76]. However, the effect of cell density on cell delivery has not been studied. We therefore compared efficiency of cell infusion at two clinically used cell densities. Efficiency of live-cell infusion and cell viability both decreased at a higher cell density, while efficiency of dead-cell infusion increased at a higher density and exceeded 100%. Higher cell viability has also been reported at higher cell densities in rat BMSC [50]. In contrast however, Zhang et al. reported that a higher cell density accelerated lactate accumulation and increased late-stage apoptosis in hADSC [47]. Differences in efficiency of cell infusion at different cell densities may thus be related to density-dependent cell death, due to accelerated nutrient deprivation and the accumulation of waste products. In contrast, supplementing high-density cADSC with AS increased efficiency of dead-cell infusion, but also increased of live-cell infusion and cell viability. AS supplementation increased the number of dead cells as at low cell density, but the relative viability also increased, because the reduction in cell adhesion to the apparatus significantly increased efficiency of live-cell infusion. This may be attributed to the more pronounced adhesion-inhibiting effect of AS supplementation, given that a higher cell density increases the chance of cell contact with and thus subsequent adhesion to the apparatus wall. These results suggest that cells should be diluted to the lowest possible density to improve delivery, while serum or albumin supplementation may be effective if a higher cell density is necessary.

Considering the results of all the above experiments, we considered that the optimal infusion procedure involved suspending cADSC in NS supplemented with 5% AS at a density of 5 × 10^5^ cells/mL, and infusing them using an automatic infusion device for 15 min, resulting in the highest efficiency of live-cell infusion. This procedure is therefore recommended as the standard procedure for intravenous administration of ADSC to patients in terms of cell-delivery efficiency. AS supplementation did not significantly improve efficiency of live-cell infusion, but procedures with a low cell density and short infusion duration would result in fewer adherent cells, suggesting that the benefits of AS supplementation were not fully realized. However, if the infusion volume or rate is limited due to the patient’s underlying disease, such as cardiac disease, the infusion time needs to be longer or the cell density higher, in which case the use of DEX or serum supplementation may be effective.

## 4. Materials and Methods

### 4.1. Study Design

**Figure 9:**
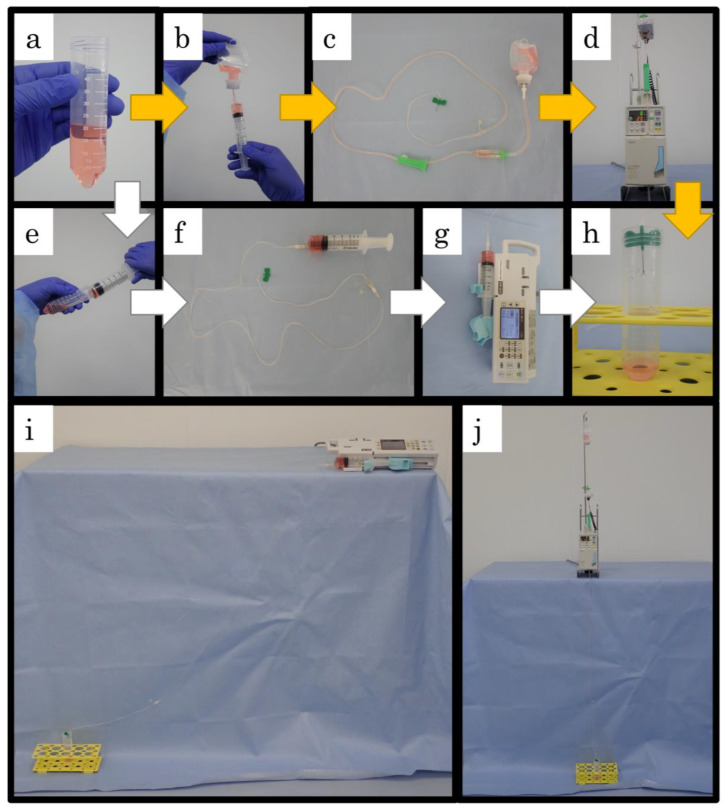
Outline the infusion procedures. (**a**), Suspend 1 × 10^7^ cADSC in 20 mL NS to prepare a cell suspension with a density of 5 × 10^5^ cells/mL; (**b**), The prepared solution is collected in a 25 mL syringe with an 18G needle and transferred to an empty 50 mL infusion bag; (**c**), The cell-containing bag is connected to the infusion tube with a 21G winged needle; (**d**), The bag is placed in a drop-controlled automatic infusion device; (**e**), The prepared solution is collected directly in a 50 mL syringe with an 18G needle; (**f**), the cell-filled syringe is connected to an extension tube with a 21G winged needle; (**g**), the syringe was placed in a syringe pump; (**h**), The winged needle tip is placed in a conical tube and the infused cells were collected. The conical tubes are replaced every 15 min, mixed immediately by gentle inversion; (**i**), Overall view of an infusion using a syringe pump. The conical tube for collecting the flowing cell suspension is placed lower than the syringe pump and the extension tube is not deflected; (**j**), Overall view of an infusion using an infusion device. The cell suspensions in this series of photographs are stained red for photography to visualize suspension transfer. Yellow arrow: infusion procedure using an infusion device; White arrow: infusion procedure using a syringe pump.

To establish the optimal MSC infusion procedure, we examined five factors: infusion apparatuses, suspension solutions, serum supplementations, infusion time and rates, and cell densities. The number of 1 × 10^7^ live-cells was suspended in 20 mL of NS to a density of 5 × 10^5^ cells/mL (Figure 9a), and then cADSC suspended in NS were filled into 50 mL infusion bag (Otsuka Pharmaceutical, Tokyo, Japan) (Figure 9b) or 50 mL eccentric luer slip syringe (Terumo, Tokyo, Japan) (Figure 9e). The cell-containing bag was connected to the infusion apparatus with a 21G winged needle (Figure 9c), while the cell-filled syringe was connected to an extension tube with a 21G winged needle (Figure 9f). The bag was then placed in a drop-controlled automatic infusion device (Figure 9d) and the syringe was placed in a syringe pump (Figure 9g). The basal infusing condition was at a rate of 0.33 mL/min for 60 min, and infused cells were collected directly into conical tubes (Figure 9h).

Experiment 1 was performed to compare the cell-delivery efficiencies of the different infusion apparatuses (Figure 9i,j). Efficiency of cell infusion and cell viability after infusion were measured and the apparatus showing the better delivery was applied in subsequent experiments. Experiment 2 and 3 were performed to evaluate the effect of the suspension solution (DEX and NS) and of supplementing serum as a cytoprotectant and cell-adhesion inhibitor. In experiment 4, we investigated the effect of cell infusion time and rate by flowing cADSC over 15, 30, and 60 min. Additionally, we compared the effects of infusing cell suspensions at densities of 5 × 10^5^ and 2 × 10^6^ cells/mL in experiment 5. Finally, considering the results of the above experiments, we identified the best cell-delivery conditions for the infusion of cADSC. All experimental procedures were performed at room temperature, and the infusion bags or syringes were shaken gently every 10 min. Five cADSC of different donor origin were used in each experiment (*n* = 5) and repeated in three independent experiments.

### 4.2. Isolation, Culture and Cryopreservation of cADSC

Adipose tissue was collected aseptically from the falciform ligament fat of five healthy dogs under general anesthesia. The animal study protocol was approved by the Bioethics Committee of Nippon Veterinary and Life Science University; approval number 2021S-40, 8 September 2021. The dogs were handled according to the animal care guidelines of the Institute of Laboratory Animal Resources, Nippon Veterinary and Life Science University, Japan. The collected adipose tissue was washed in phosphate-buffered saline (PBS), minced, and digested with collagenase type I (Sigma-Aldrich, St. Louis, MO, USA) at 37 °C for 45 min with intermittent shaking. After washing with PBS and centrifuging at 1400× *g* for 5 min at room temperature, the pellets containing the stromal vascular fraction were resuspended, filtered through a 100-μm nylon mesh, and incubated overnight in high glucose Dulbecco’s Modified Eagle’s Medium (DMEM) supplemented with 10% fetal bovine serum (FBS; Capricorn, Hessen, Germany) and a 1% antibiotic-antimycotic solution (Thermo Fisher Scientific, Waltham, MA, USA) in a humidified atmosphere with 5% CO_2_ at 37 °C. Non-adherent cells were removed by aspiration of the culture supernatant, and adherent cells were washed twice with PBS. The medium was then changed every 3–4 days. The cells were detached using trypsin-EDTA solution (Sigma-Aldrich) when they reached 80–90% confluence, and cryopreserved or passaged. Cells were suspended in 1 mL of commercially available serum-free cell-preservation solution (Takara Bio, Shiga, Japan) at 1–2 × 10^6^ cells in passages 1 or 2 and cryopreserved in liquid nitrogen until experimentation.

### 4.3. Characterization of cADSC Surface Markers

Phenotype analysis was performed as described previously [77]. Passage 2 cADSC were placed in fluorescence-activated cell sorting (FACS) tubes (BD Biosciences, Franklin Lake, NJ, USA; 2 × 10^5^ cells/tube), washed with FACS buffer (PBS containing 2% FBS), followed by blocking Fc receptors with canine Fc receptor binding inhibitor (Thermo Fisher Scientific), and then incubated on ice for 20 min with the following fluorescein (FITC)- or phycoerythrin (PE)-conjugated antibodies: anti-CD14-FITC (BD Pharmingen, San Diego, CA, USA), anti-CD29-PE (BioLegend, San Diego, CA, USA), anti-CD34-PE (R&D Systems, Minneapolis, MN, USA), anti-CD44-PE (BioLegend), anti-CD45-FITC (eBioscience, San Diego, CA, USA), and anti-CD90-PE (eBioscience) or their respective isotype controls. The cells were washed twice with FACS buffer and resuspended in 500 μL FACS buffer. Fluorescence was evaluated by flow cytometry in a CytoFLEX instrument (Beckman Coulter, Brea, CA, USA). The data were analyzed using CytExpert ver2.0 analysis software.

### 4.4. Differentiation Assay

For adipogenic differentiation, passage 2 cADSC were seeded on 12-well plates at a concentration 4 × 10^4^ cells/well and cultured in DMEM supplemented with 10% FBS and 1% antibiotic-antimycotic solution until 80% confluency. The medium was then replaced with 1 mL of adipogenesis differentiation medium (Thermo Fisher Scientific) and changed every 3 days. Adipogenesis was analyzed by Oil Red O staining after 21 days.

For osteogenic differentiation, passage 2 cADSC were seeded on 12-well plates at a concentration 2 × 10^5^ cells/well and incubated in DMEM supplemented with 10% FBS and 1% antibiotic-antimycotic solution for 24 h. The medium was then replaced with 1 mL of osteogenesis differentiation medium (Thermo Fisher Scientific) and changed every 3 days. For osteogenic analysis, mineral deposits were analyzed quantitatively by Alizarin Red staining after 21 days.

For chondrogenic differentiation, 2 × 10^5^ passage 2 cADSC were suspended in 1 mL of chondrogenic differentiation medium (STEMCELL Technologies, Vancouver, BC, Canada) and 0.5 mL was aliquoted into a 15 mL conical tube and then centrifuged at 300× *g* for 5 min. The lid was then loosened and the cells were incubated for 3 days, after which 0.5 mL of medium was added and replaced every 3 days. Chondrogenic differentiation was evaluated by Alcian Blue staining after 21 days.

### 4.5. Collection of Canine AS

Peripheral venous blood (10 mL) was collected aseptically from the jugular vein of five beagles (different from ADSCs donors used in the experiment) under mild sedation, by a veterinarian using a 10 mL syringe with a 23G needle. The collected blood was centrifuged at 1700× *g* for 15 min and the separated serum was frozen at –80 °C for subsequent experiments. Prior to use, the serum was thawed rapidly in a thermostatic chamber at 37 °C.

### 4.6. Preparation of cADSC

Passage 1 or 2 cryopreserved cADSC were thawed, incubated, and harvested at 80–90% confluency for experiments. The harvested cells were washed with PBS and suspended in 10 mL of NS or DEX, followed by filtration through a 70-μm nylon mesh. The numbers of live and dead cells were then measured manually using the Trypan Blue staining method. The cell suspension was then aliquoted to obtain the required number of cells and diluted with the corresponding solution to a final volume of 20 mL, supplemented with 5% AS if necessary. The prepared solution was collected in a 25 mL syringe with an 18G needle and transferred to an empty 50 mL infusion bag or collected directly in a 50 mL syringe with an 18G needle. This series of preparatory procedures took approximately 30–40 min.

### 4.7. Efficiency of Cell Infusion and Cell Viability

During cell infusion, the needle tip was placed in a conical tube and the infused cells were collected. The conical tubes were replaced every 15 min, mixed immediately by gentle inversion, and the number of unstained viable cells and stained dead cells were counted manually by Trypan Blue staining.

The efficiency of live-cell infusion (%) was calculated by dividing the number of live cells collected after infusion by the number of live cells before infusion (1.0 × 10^7^) and multiplying by 100. The efficiency of dead-cell infusion (%) was calculated by dividing the number of dead cells collected after infusion by the number of dead cells contained cell suspension before infusion and multiplying by 100. Cell viability (%) was calculated by dividing the number of live cells by total number of live and dead cells at each time point before and after infusion and multiplying by 100.

The effects of the suspension solution alone and combined with AS on cell viability at room temperature were determined by nuclear staining with 7-AAD (BioLegend). cADSC were suspended in NS, DEX, NS supplemented with 5% AS (NS + AS), and DEX supplemented with 5% AS (DEX + AS) using the same preparation procedure described above, and 500 μL of the respective cell suspensions were placed in FACS tubes (1 × 10^6^ cells/tube) and allowed to stand at room temperature for 20 min, followed by the addition of 5 μL of 7-AAD to each FACS tube and incubation at room temperature for 10 min. Cell death was determined by flow cytometry in a CytoFLEX instrument. Data were analyzed using CytExpert ver2.0 analysis software.

### 4.8. Statistical Analysis

All data are presented as the mean ± standard error. Differences between two groups were analyzed using Welch’s *t*-test and differences among multiple groups were assessed by one-way analysis of variance and compared using the Tukey–Kramer post hoc test. *p* < 0.05 was considered statistically significant. Statistical analyses were performed using R commander 4.1.2.

## 5. Conclusions

The results of this study demonstrated that the highest cell-delivery efficiency was achieved when canine ADSCs were suspended in NS with 5% AS at a density of 5 × 10^5^ cells/mL, and infused using an automatic infusion device for 15 min. This procedure is accordingly recommended as the standard procedure for the intravenous administration of ADSCs to patients in terms of cell-delivery efficiency. Further studies are needed to determine the effect of this injection method on the long-term survival and function of ADSCs to ensure their therapeutic potential in vivo.

## Data Availability

The data supporting the findings of this study are available from the corresponding author upon reasonable request.
